# Associations of Genetically Predicted NPR3 and NPR2 Perturbation and Preeclampsia Risk: A Two-Sample Mendelian Randomization Analysis

**DOI:** 10.1155/ijhy/9972031

**Published:** 2025-05-15

**Authors:** Roxane de La Harpe, Tormod Rogne, Michael Nyberg, Héléne T. Cronjé, Stephen Burgess, Ville Karhunen, Dipender Gill

**Affiliations:** ^1^Department of Medicine, University Hospital of Lausanne, Lausanne, Vaud, Switzerland; ^2^Department of Community Medicine and Global Health, University of Oslo, Oslo, Norway; ^3^Department of Chronic Disease Epidemiology, School of Public Health, New Haven, Connecticut, USA; ^4^Research and Early Development, Novo Nordisk A/S, Novo Nordisk Park, Måløvc, Region Hovedstaden, Denmark; ^5^MRC Biostatistics Unit, School of Clinical Medicine, University of Cambridge, Cambridge, UK; ^6^Department of Epidemiology and Biostatistics, School of Public Health, Imperial College London, London, UK

## Abstract

**Background:** Preeclampsia, a pregnancy complication marked by hypertension after 20 weeks of gestation, arises from placental factors that impair maternal vascular function. C-type natriuretic peptide (CNP), known for its vasodilatory role, may help counter preeclampsia-related vascular dysfunction. This study aimed to explore the effect of CNP on preeclampsia risk using the Mendelian randomization (MR) framework.

**Methods:** Genetic instrumental variables that mimic the effects of CNP signaling (through natriuretic peptide receptor 2 [NPR2] activation or reduced NPR3-mediated clearance) were identified in the genes encoding the two receptors. This discovery emerged from a multiancestry genome-wide association study (GWAS) involving over 5 million individuals. Female-specific genetic association estimates were obtained from individual-level data comprising 198,402 female participants in the UK Biobank. Two-sample MR analyses were conducted to investigate the effects of NPR2 activation and NPR3 function on preeclampsia, utilizing the largest publicly available GWAS on preeclampsia, which included 296,824 female participants.

**Results:** Genetically proxied reduced NPR3 function was associated with a lower risk of preeclampsia (odds ratio (OR): 0.46, 95% confidence interval 0.30–0.69). In contrast, genetically proxied increased NPR2 activation lacked significant association, likely due to underpowered genetic instruments. Sensitivity analyses indicated robust findings with minimal pleiotropy, meaning the genetic variants used primarily influenced preeclampsia through the intended biological pathway rather than affecting multiple unrelated traits.

**Conclusion:** This study employed the MR paradigm to provide genetic evidence supporting the protective effects of CNP (through reduced NPR3 function) on the risk of preeclampsia. However, it is important to gather additional evidence from other sources before moving forward with clinical development efforts to explore CNP as a potential treatment for preeclampsia.

## 1. Introduction

Preeclampsia is a significant obstetric complication affecting approximately 2%–4% of pregnancies worldwide, contributing to substantial maternal and neonatal morbidity and mortality [1, 2]. It is characterized by new-onset hypertension after 20 weeks of gestation, often accompanied by proteinuria or signs of end-organ dysfunction, resulting from placental insufficiency and widespread maternal vascular dysfunction [1, 3].

The pathophysiology of preeclampsia is complex and not yet fully understood, which limits the development of targeted therapies. However, it is primarily a placental disorder, with placental dysfunction playing a central role in disease onset and progression. This dysfunction is particularly linked to inadequate modification of maternal spiral arteries by trophoblastic cells from the placenta [2, 3]. Another key pathogenic mechanism involves the release of antiangiogenic and proinflammatory factors from the ischemic, dysfunctional placenta into the maternal circulation, leading to widespread endothelial dysfunction and vascular dysregulation [2, 4]. Impaired maternal systemic arterial compliance and increased peripheral vascular resistance contribute to hypertension and organ hypoperfusion [4]. Given this, targeting persistent systemic vasoconstriction and improving endothelial function in the maternal vasculature may be key strategies for effectively managing preeclampsia.

Currently, there is no disease-modifying therapy for established preeclampsia, and the only definitive cure remains delivery, which is often required when maternal or fetal health is at risk [2]. Existing treatment strategies emphasize prevention and symptom management, including promoting physical activity, administering low-dose aspirin, calcium supplementation, controlling blood pressure, and using magnesium sulfate to prevent seizures in severe cases [2].

Numerous clinical trials are exploring targeted therapies that address the underlying pathogenesis of preeclampsia [2]. These include plasmapheresis to remove antiangiogenic factors, monoclonal antibodies targeting tumor necrosis factor-α or complement pathways, and gene-silencing strategies designed to reduce antiangiogenic factor production or angiotensinogen expression [2]. These innovative approaches hold promise for improving outcomes in preeclampsia by addressing its root causes rather than solely managing symptoms.

C-type natriuretic peptide (CNP) is an endothelial-derived peptide that plays a key role in vascular homeostasis through its vasodilatory properties [5, 6]. Research on CNP in pregnancy is limited, but studies indicate that circulating levels of CNP generally remain stable throughout most of an uncomplicated pregnancy but may decline between 22 and 40 weeks [7]. Small observational studies [8–10] have shown elevated circulating levels of NT-proCNP during mid-to-late gestation prior to the onset of preeclampsia, whereas another study found no elevation of CNP levels [11]. Several studies have demonstrated CNP's therapeutic potential for hypertensive disorders in nonpregnant conditions. References [4, 12, 13] these findings may suggest a compensatory mechanism aimed at counteracting the vasoconstriction associated with preeclampsia by increasing the expression of the CNP precursor. This raises also the question of whether preeclampsia might affect the conversion of the CNP precursor into its active form [4]. Recently, Fato et al. demonstrated in an experimental study that CNP induces vasorelaxation in arteries from pregnant patients with preeclamptic vasoconstriction, primarily through the activation of natriuretic peptide receptor 2 (NPR2 or NPR-B) [4]. NPR2 and NPR1 (or NPR-A) activate cGMP production and mediate the physiological effects of natriuretic peptides, with NPR1 being more involved with atrial natriuretic peptide (ANP) and brain natriuretic peptide (BNP) and NPR2 mainly responding to CNP [14]. NPR3 (or NPR-C) is primarily a clearance receptor, regulating the levels of all natriuretic peptides in the circulation [14].

Building on the promising findings from Fato et al. and the limited availability of disease-modifying therapies for preeclampsia, we aimed to contribute to the growing body of evidence supporting CNP as a potential drug target for preeclampsia. Specifically, we seek to provide genetic evidence to support this hypothesis, which has not been explored to date. In our study, we employed a drug-target Mendelian randomization (MR) approach, leveraging genetic variants that proxy CNP agonist action to investigate its potential protective effects against preeclampsia.

## 2. Methods

### 2.1. Study Overview

Genetic variants predicting the perturbation of pharmacological targets can be used in the MR paradigm to rapidly and cost-effectively investigate on-target drug effects [15]. The random allocation of genetic variants at conception means that this approach is less vulnerable to bias from environmental confounding and reverse causation that can hinder causal inference in traditional epidemiological studies.

CNP also promotes longitudinal bone growth through NPR2-mediated activation. As a result, height may increase with enhanced NPR2 activation or reduced NPR3 function, as NPR3 serves to clear all three types of natriuretic peptides. Hence, we inferred that genetic variants associated with increased height, located in the genes encoding *NPR2* and *NPR3* receptors, can serve as proxies for the biological pathway of interest (i.e., CNP agonism action). These genetic variants were then used within the MR paradigm for studying the effect of CNP through increased NPR2 activation and reduced NPR3 function on the risk of preeclampsia.

MR is a statistical approach that uses genetic variants as instrumental variables to investigate the causal effects of an exposure on an outcome of interest. There are three main assumptions underlying this approach. Firstly, the genetic instruments must be strongly associated with the exposure of interest (i.e., the relevance assumption). Secondly, no confounding factors influence the association between the genetic instruments and the outcome of interest (i.e., the independence assumption). Thirdly, the genetic instruments are only related to the outcome via the exposure, ensuring the absence of pleiotropic effects that may bias MR estimates (i.e., the exclusion restriction assumption) [16].

**Figure 1:**
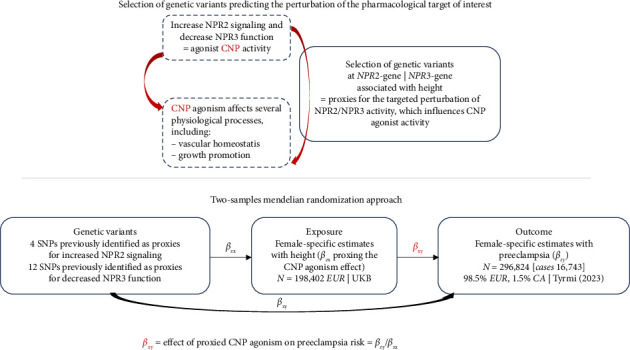
Schematic overview of study design.

The study overview is schematically depicted in Figure 1.

### 2.2. Genetic Instrument Selection

We used, respectively, 4 and 12 previously identified single-nucleotide polymorphisms (SNPs) associated with increased height at the *NPR2* and *NPR3* gene regions at *p* < 5 × 10^−8^ (pruning to pairwise *r*^2^=0.1, using the 1000Genomes European reference panel) as genetic instruments for pharmacological perturbation of these targets. These variants were identified by Cronjé et al. using a multiancestry GWAS on height in over 5 million individuals [17]. Details on the identification of the SNPs, including their location, effect allele, original effect size, standard error, and F-statistics, can be found in the original study. We would like to clarify again that, while height is used as the nominal exposure for selecting variants at the *NPR2* and *NPR3* gene regions and for scaling our estimates, height should be considered as a biomarker serving as a proxy for the drug-target effect being assessed (i.e., the perturbation of NPR2/NPR3 activity leading to increased CNP agonist-induced effects), rather than as the causal risk factor.

### 2.3. Female-Specific Estimates of SNPs Proxying CNP Agonism

We obtained female-specific genetic association estimates between the selected e SNPs and height using individual-level data of female participants of the UK Biobank, who had both height measurements and genetic data available. UK Biobank is a large, ongoing, prospective cohort study, which recruited 502,713 people (intended to be aged 40–69 years, mean age 56.5 years, 45.6% men) from 2006 to 2010 in England, Scotland, and Wales, 94% of whom self-reported European ancestry. Genotyping was assessed using the axiom array [18].

For quality control, participants were excluded if they fulfilled the following criteria: (1) have excess relatedness (more than 10 putative third-degree relatives); (2) have inconsistent information about sex based on genotyping and self-report; (3) have sex chromosomes not XX or XY; (4) have poor-quality genotyping based on heterozygosity and missing rates; or (5) have withdrawn from UK Biobank.

After quality control, 198,402 female participants who have been identified had height measurement and genetic data available. We obtained the sex-specific unit difference in height (cm) per effect allele for each selected SNP using linear regressions controlling for participants' age, assay array, and the 10 principal components to account for the population structure.  Table 1 presents the final estimates for each SNP.

### 2.4. Female-Specific Summary Statistics on Preeclampsia

Summary statistics for obtaining genetic association estimates for preeclampsia were downloaded from the largest publicly available genome-wide association study (GWAS) conducted by Tyrmi et al. [19] This GWAS was a meta-analysis of data from the Finnish Genetics of Pre-eclampsia Consortium (FINNPEC), FinnGen, and the Estonian Biobank. It also incorporated summary statistics from a prior meta-analysis of preeclampsia conducted by Steinthorsdottir et al. [20] The mean (SD) age in each cohort was 30.3 [5.5], 28.7 [5.6], 29.7 [7.0], and 28 [not available] years, respectively. The pooled sample consisted of 296,824 females, primarily of European ancestry, including 16,743 cases, of which 2296 (13.7%) were of Central Asian ancestry, and 280,081 controls, with 2059 (0.7%) from Central Asian ancestry. Genotyping was performed using Illumina and Affymetrix arrays, and estimates were adjusted by age, genotyping batches, and the first 10 PCs.

### 2.5. Statistical Analysis

We conducted two-sample random-effect inverse-variance weighted (IVW) MR [21] as the main analysis to estimate the association of genetically predicted CNP agonism (through *NPR2*- and *NPR3*-genetically predicted height) on preeclampsia risk, accounting for the correlation between SNPs (Figure 1).

We also assessed instrument strength with the F-statistic (F-statistic > 20 minimize the risk of weak instrument bias). Heterogeneity in the SNP-specific effects was assessed with Cochran's Q test. For sensitivity analyses, we used the MR-Egger and weighted median (WM) methods, as they make different assumptions about the presence of invalid instruments and pleiotropy. Additionally, we conducted a leave-one-out analysis to assess whether any MR association was driven by a single pleiotropic SNP that violates the requisite modeling assumptions.

Given the provided sample size, we calculated the statistical power of our analysis for each instrumental variable (i.e., NPR2 and NPR3) to detect a true odds ratio (OR) of 0.6. This calculation was based on a total sample size of 296,824, with 5.6% of cases and variances of 0.0004 for NPR2 and 0.002 for NPR3, utilizing formulas from Brion et al. [22] The statistical power for NPR3 was 66%, while it was only 18% for NPR2. Since NPR2 was identified as the primary pathway for the CNP effect in the study by Fato et al., we decided to proceed with the analyses using this instrument despite its lower power.

Analyses were performed in R (version 4.3.1) using the TwoSampleMR and MendelianRandomization packages.

## 3. Results

The F-statistics for the NPR2 and NPR3 instruments were 17.6 and 35.4, respectively.

**Figure 2:**
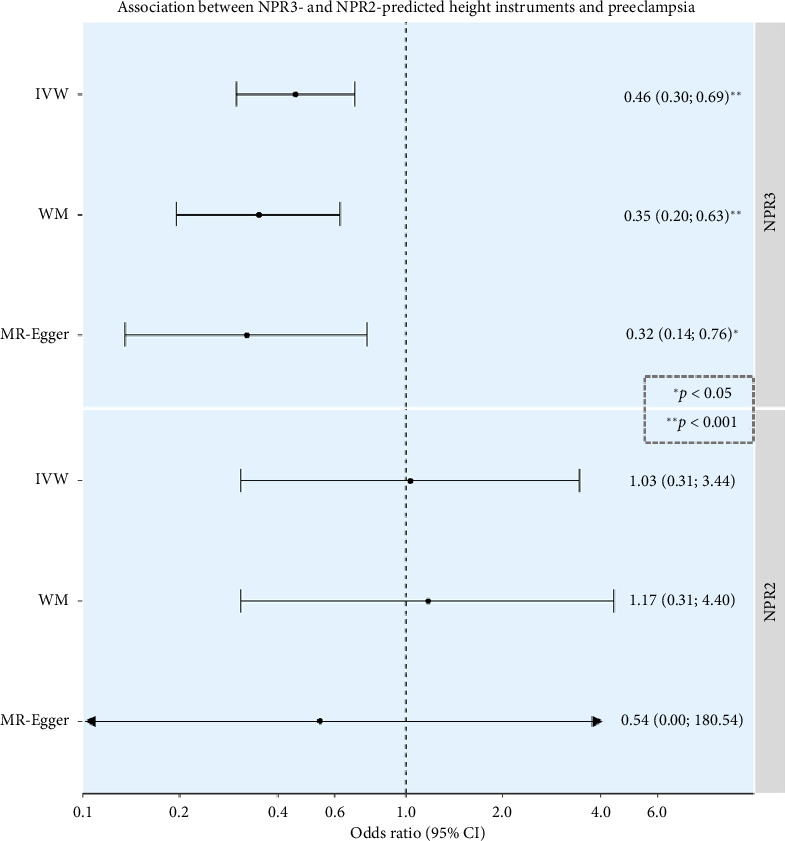
Study results.

A 1-SD increase in *NPR3*-predicted height was associated with a 54% reduction in the odds of preeclampsia (OR 0.46, 95% confidence interval [CI] 0.30–0.69) (Figure 2). No evidence for an association between *NPR2*-predicted height variants and preeclampsia was observed.

Sensitivity analyses indicated no strong evidence of heterogeneity *p* value = 0.55 for NPR3 association and 0.26 for NPR2 association or pleiotropy (*p* value for MR-Egger intercept = 0.67 for NPR3 association and 0.84 for NPR2 association), with consistent estimates providing evidence of an association. No outlier variant was found to drive the estimates in leave-one-out analyses, with OR point estimates ranging from 0.32 to 0.47 for NPR3 and from 0.72 to 1.56 for NPR2, with all confidence intervals overlapping.

## 4. Discussion

Preeclampsia is a serious pregnancy complication for which there are currently no disease-modifying therapies. The genetic instrument that proxies CNP signaling through reduced NPR3 clearance function suggests a potential causal relationship between CNP and preeclampsia, reinforcing the findings of Fato et al. of CNP's involvement in the pathophysiology of preeclampsia [4]. The wide confidence interval of our result with the genetic instrument that proxies CNP signaling through increased NPR2 activity suggests that this instrumental variable may have been indeed underpowered to detect an effect.

CNP has been explored as a therapeutic agent to promote vasodilation and improve blood pressure in nonpregnant conditions, given its widespread expression in endothelial cells and its diverse roles in the vasculature. These roles include regulating vascular tone, modulating leukocyte activation, promoting angiogenesis, and facilitating smooth muscle and endothelial cell proliferation, all of which contribute to maintaining vascular integrity [4]. Human studies on CNP demonstrated that CNP infusions lead to a reduction in blood pressure [23, 24]. Additionally, animal studies have shown that endothelial CNP deletions are associated with an increase in blood pressure, especially among women [25]. Recently Fato et al. demonstrated that CNP can vasodilate omental arteries from pregnant patients that are constricted with serum from patients with early-onset preterm preeclampsia [4]. Our results from the MR paradigm provide genetic evidence supporting the protective effect of CNP signaling through reduced NPR3 function in preeclampsia. To our knowledge, there are no other MR studies that have addressed this similar research question. However, the largest and most recent GWAS by Tyrmi et al. also highlighted the *NPR3* gene locus, which is in proximity to genes involved in the remodeling of uterine spiral arteries, as a gene candidate for maternal hypertensive disorders [19]. The strength of this study lies in its use of the MR paradigm, which reduces susceptibility to environmental confounding and reverse causation bias. Furthermore, by incorporating genetic association summary data, we employ a human-centric analysis that yields findings more directly translatable to clinical applications, thereby addressing some of the limitations associated with animal models in drug-target development. However, triangulation with other forms of evidence that operate under different assumptions, such as large cohort studies or nested case-control studies, along with experimental investigations into CNP regulation in the placenta and placental diseases, is essential before translating these findings into clinical applications.

A limitation of our approach is that the available data predominantly represent European cohorts, which may limit the generalizability of our findings to other ancestry groups. However, this homogeneous ancestry distribution can help mitigate bias arising from differences in population structures, such as variations in allele frequencies among subpopulations between the two samples used in the MR analyses. Additionally, genetic estimates were adjusted for the first 10 genetic components, further reducing the potential for bias. In addition, our findings are constrained by the possibility of genetic confounding due to unknown pleiotropic effects of the genetic variants used as instruments. While we were able to test the first assumption that our instrumental variables are strongly associated with the exposure, evidenced by an F-statistic greater than 20 for NPR3 instrument, the other two assumptions cannot be specifically tested. Our sensitivity analyses did not indicate a pleiotropic effect, suggesting that our genetic variants do not indirectly influence the outcome through another risk factor, which could introduce confounding between the exposure and the outcome. However, we cannot definitively rule out this possibility. Additionally, we cannot completely rule out the possibility that our association was influenced by confounding due to linkage disequilibrium, where another SNP in linkage disequilibrium directly affects the outcome or a confounding pathway. Nonetheless, this concern is mitigated by the pruning process used for selecting SNPs and the adjustments made in the analysis to account for residual correlation. Lastly, our method only captures the potential effects of NPR2 and NPR3 on preeclampsia risk that are related to the same signaling pathways affecting height. If these proteins influence preeclampsia risk through different mechanisms, our instruments may not detect these effects. Given the roles of ANP and BNP in blood pressure regulation and their suggested associations to gestational hypertension [26], the observed effects of reduced NPR3 function on preeclampsia risk may also involve enhanced signaling by these peptides.

## 5. Conclusion

This study employed the MR paradigm to contribute the body of evidence suggesting a potential beneficial effect of CNP signaling in preeclampsia. However, it is important to gather additional evidence from other sources before moving forward with clinical development efforts to explore CNP as a potential treatment for preeclampsia, a condition that significantly contributes to maternal morbidity and mortality.

Full descriptions of the populations analyzed in the GWASs from which summary data were obtained for analyses are available in the original publications. Abbreviation: CA, Central Asian ancestry; EU, European ancestry; UKB, UK Biobank.

Results, based on 12 SNPs at the *NPR3* gene and 4 SNPs at the *NPR2* gene, include IVW MR estimates, along with sensitivity analyses using the WM and MR-Egger methods. OR estimates are scaled per 1-SD increase in height. Full descriptions of the populations analyzed in the GWASs from which summary data were obtained for analyses are available in the original publications. An asterisk denotes *p* value < 0.05, and two asterisks denote *p* value < 0.001.

## Figures and Tables

**Table 1:** Instrument SNPs used for NPR2- and NPR3-predicted height.

SNP	Chromosome	Effect allele	Other allele	EAF	Beta	se	*p* value	MAF	R^2^	F stat
rs1471154	5	C	T	0.885826	0.014583	000.486	0.002696	0.114174	4.54E − 05	9.002612
rs16890002	5	C	G	0.051633	−0.01764	0.006963	0.011271	0.051633	3.24E − 05	6.422206
rs17540044	5	C	G	0.255109	0.031949	0.003542	1.9E − 19	0.255109	000.041	81.36019
rs976576	5	C	T	0.748926	−0.00782	0.003549	0.027605	0.251074	2.45E − 05	4.852673
rs696831	5	A	G	029.189	−0.00443	000.359	0.217335	029.189	7.67E − 06	1.521885
rs1060559	5	T	C	0.109257	0.011892	000.496	0.016511	0.109257	2.9E − 05	5.747696
rs3792752	5	G	A	0.258647	0.036183	000.352	8.81E − 25	0.258647	0.000532	105.6764
rs1173771	5	G	A	060.039	−0.03549	0.003148	1.75E − 29	039.961	0.000641	127.1622
rs9292469	5	T	C	0.343028	−0.01514	0.003257	3.33E − 06	0.343028	0.000109	21.61895
rs7722828	5	T	C	0.078798	0.023895	0.005738	3.12E − 05	0.078798	8.74E − 05	17.34245
rs1472261	5	A	G	0.117881	−0.00763	0.004792	011.138	0.117881	1.28E − 05	2.534553
rs2331101	5	T	C	0.252945	−0.0225	0.003545	2.23E − 10	0.252945	0.000203	40.26129
rs867194	9	G	A	0.276416	0.012901	0.003446	0.000182	0.276416	7.06E − 05	14.01364
rs7847621	9	G	A	0.614089	0.018871	0.003168	2.58E − 09	0.385911	0.000179	35.48238
rs4879930	9	T	C	072.938	−0.00937	0.003474	0.007025	027.062	3.66E − 05	7.266677
rs12000024	9	G	A	0.232854	0.013522	0.003647	0.000209	0.232854	6.93E − 05	13.74841

*Note:* beta: unit difference in height (cm) per effect allele; se: standard error of beta; *R*^2^: the proportion of the variance in height explained by the SNP [2 × EAF × (1 − EAF) × beta2]; F statistic: a product of the size and precision of the genetic association [*R*^2^ × (*N* − 2)/(1 − *R*^2^)]; SNP: single-nucleotide polymorphism.

Abbreviation: EAF, effect allele frequency.

## Data Availability

Publicly available genome-wide association study (GWAS) summary data, used in this work, can be obtained from the Catalog of human genome-wide association studies (https://www.ebi.ac.uk/gwas/home). Other sex-specific data described in the manuscript will be available upon request and approval by the UK Biobank. The code used for this work may be obtained on request from the corresponding author.
